# On-call emergency workload of a general surgical team

**DOI:** 10.4103/0974-2700.44677

**Published:** 2009

**Authors:** Masood Jawaid, Syed Muhammad Raza, Shams Nadeem Alam, S Manzar

**Affiliations:** Surgical Unit II, Civil Hospital Karachi, Pakistan

**Keywords:** Appendicitis, emergency surgery, on-call, pattern, training, trauma, work load

## Abstract

**Background:**

To examine the on-call emergency workload of a general surgical team at a tertiary care teaching hospital to guide planning and provision of better surgical services.

**Patients and Methods:**

During six months period from August to January 2007; all emergency calls attended by general surgical team of Surgical Unit II in Accident and Emergency department (A and E) and in other units of Civil, Hospital Karachi, Pakistan were prospectively recorded. Data recorded includes timing of call, diagnosis, operation performed and outcome apart from demography.

**Results:**

Total 456 patients (326 males and 130 females) were attended by on-call general surgery team during 30 emergency days. Most of the calls, 191 (41.9%) were received from 8 am to 5 pm. 224 (49.1%) calls were of abdominal pain, with acute appendicitis being the most common specific pathology in 41 (9.0%) patients. Total 73 (16.0%) calls were received for trauma. Total 131 (28.7%) patients were admitted in the surgical unit for urgent operation or observation while 212 (46.5%) patients were discharged from A and E. 92 (20.1%) patients were referred to other units with medical referral accounts for 45 (9.8%) patients. Total 104 (22.8%) emergency surgeries were done and the most common procedure performed was appendicectomy in 34 (32.7%) patients.

**Conclusion:**

Major workload of on-call surgical emergency team is dealing with the acute conditions of abdomen. However, significant proportion of patients are suffering from other conditions including trauma that require a holistic approach to care and a wide range of skills and experience. These results have important implications in future healthcare planning and for the better training of general surgical residents.

## INTRODUCTION

General surgery is a major specialty dealing with high volumes of emergency admissions, their management and a very wide range of elective procedures.[1] Emergency surgical care is of high priority in tertiary care teaching hospitals and it is a very important aspect of training surgical residents. There is a continuous increase in the number of emergency admissions[2] which include surgical emergencies.[3] Many studies have observed the pattern of emergency surgical admissions[4,5] and emergency surgeries.[6] However, there is little data available on spectrum of the emergency general surgical calls worldwide and literature search showed that no study has been conducted or documented regarding the on-call work-load in Pakistan. In fact, there is a greater workload of the emergency surgical team than revealed by auditing surgical admissions and operations alone.

This study was planned to observe the on-call emergency workload of a general surgical team so that some guidelines could be postulated for better patient care and more appropriate training of junior surgeons keeping in view the prevailing local acute surgical diseases patterns.

## PATIENTS AND METHODS

Civil Hospital, Karachi (CHK) is a 1670-bed tertiary care teaching hospital in the public sector that imparts both undergraduate and postgraduate teaching and training. It is one of the teaching hospitals affiliated with Dow University of Health Sciences (DUHS). CHK attracts patients not only from Karachi but also from the rural areas of the Sindh and Balochistan provinces. The department of surgery comprises six general surgical units besides the specialties of neurosurgery, paediatric surgery, orthopedics, urology, vascular, maxillo-facial, and plastic surgery. All units function independently. Surgical Unit II, in which this study was conducted, has one on-call emergency day in a week and every sixth on-call emergency Sunday on rotation. On each 24 hour of emergency call day, the surgical on-call team consisted of a consultant surgeon, four postgraduate trainees, and four house officers. This team deals with all emergency calls as well as in-patient management of the unit for elective surgery. The Emergency Operation Theatre is available 24 hours a day, fully staffed for emergency surgery.

This prospective study included all emergency calls attended by the team of Surgical Unit II in Accident and Emergency department as well as in different units of CHK on the emergency day from August 2006 to January 2007. The data was collected by on-duty surgical residents in a specially designed proforma which included timing of call, presenting complains, diagnosis, surgery (if done) and outcome apart from patient's demography. Vascular, urological, neurosurgical, orthopaedic, and paediatric surgical emergencies were excluded as there are separate teams which deal with these patients.

## RESULTS

During the study period, there were 30 emergency days. Total 456 calls were attended with 15.2 mean emergency calls per day. Mean age of patients was 35.91±16.6 years with 326 (71.5%) males and 130 (28.5) females. Most of the calls, 191 (41.9%) were received from 8 am to 5 pm [Table 1]. Out of total, 224 (49.1%) calls were of abdominal pain, with acute appendicitis being the most common specific pathology in 41 (9.0%) patients. 73 (16.0%) calls were received for trauma [Table 2]. Apart from Accident and Emergency department, 40 (8.8%) calls were received from other units. Total 131 (28.7%) patients were admitted in the surgical unit for urgent operation or observation while 212 (46.5%) patients were discharged from Accident and Emergency (A and E) department. 92 patients were referred to other units with medical referral accounts for 45 (9.8%) patients [Table 3].

**Table 1:** Timing of emergency call

Time	Number of calls	%
08:00-17:00 h	191	41.9
17:00-22:00 h	109	23.9
22:00-08:00 h	156	34.2

**Table 2:** Major disease pattern of emergency surgical call (n=456)

Disease	n	%
Abdomen pain	224	49.1
Appendicitis (acute/lump)	41	9.0
Acute cholecystitis	13	2.8
Intestinal obstruction	11	2.4
Peritonitis	9	2.0
Liver abscess	6	1.3
Perforated duodenal ulcer	4	0.9
Acute pancreatitis	4	0.9
Others with non specific abdominal pain	136	29.8
Trauma	73	16.0
Blunt abdominal trauma	21	4.6
Penetrating abdominal trauma	11	2.4
Blunt chest injury	14	3.0
Penetrating chest injury	3	0.6
Other	25	5.5
Soft tissue abscess	33	7.2
Irreducible hernias	24	5.2
Tetanus	10	2.2
Miscellaneous	92	20.1

**Table 3:** Frequency pattern of emergency call attended, referred, and outcome

Patients attended in different units	n	%
Accident and emergency	416	91.2
Medicine	22	4.8
Orthopedic	7	1.5
Neurology	5	1.1
Urology	3	0.6
Pediatric	3	0.6
**Patients referred to other units**		
Medicine	45	9.8
Urology	15	3.3
Gynecology and obstetrics	9	2.0
Orthopaedics	8	1.7
Neurosurgery	6	1.3
ENT	6	1.3
Vascular Surgery	3	0.6
**Total**	92	20.1
**Outcome of patients**		
Discharged	212	46.5
Admitted	131	28.7
For operation	104	22.8
For observation	27	5.9
Referred	92	20.1
Left against medical advice	16	3.5
Expired	5	1.1

Total 104 emergency surgeries were performed with appendicectomy being the most common procedure in 34 (32.7%) patients followed by incision and drainage 27 (26.0%) and laparotomy in 21 (20.2%) patients. All procedures performed are shown in [Table 4].

**Table 4:** Emergency operation performed during six months duration

Procedure	n	%
Appendicectomy	34	32.7
Abscess drainage/debridement	27	26.0
Laparotomy	21	20.2
Wound exploration and primary repair	9	8.6
Herniorrhaphy	7	6.7
Chest tube intubation	4	3.8
Venous Cut down	2	1.9
Total	104	100.0

## DISCUSSION

Emergency cases form the major workload of a general surgical unit. Little accurate quantitative data is available at present regarding the nature and impact of emergency surgical call workload in tertiary care public teaching hospitals. This study highlights the disease pattern encountered by an on-call team.

Results of this study showed that the majority of patients (49.1%) presented to general surgery as an emergency were suffering from acute conditions of the abdomen. It is well documented that appendicitis is the most frequent abdominal emergency worldwide.[7] In our study also, the most frequent specific diagnosis made in A and E is appendicitis.

Urgent surgical procedures were carried out in 104 (22.8%) patients, appendicectomy being the most frequent operation performed. The same trend was observed in other studies from UK[6] and Nigeria.[8] One study from Ireland[9] showed that 19.5% emergency inpatients required surgical procedures. 328 (72%) of these were performed out of the normal working hours. The commonest operations were appendicectomy (51%), abscess drainage (13%), wound toilet (13%), and laparotomy (11%).

Our study showed that majority of patients attended by an on-call general surgical team in A and E department were discharged or referred to some other units which indicates that the A and E department is not so good at filtering the patients appropriately. In our opinion, the main reason is the inadequate trained staff, both in terms of manpower and qualified emergency doctors. For all patients who presented with abdominal pain, call was given to the on-call surgical unit without doing proper resuscitation and initial work up. This increased the substantial workload on the on-call team, whose work now not only included the management of genuine surgical emergencies but also doing investigation and proper referral to other units.

The general surgeon is the captain of the trauma team. The Advanced Trauma and Life Support (ATLS) guidelines and recommendations of the Royal College of Surgeons of England state that a trauma team should include a general surgeon.[10,11] However, only a minority of all trauma patients require assessment for abdominal and/or vascular injuries by a general surgeon, with even fewer requiring surgical intervention.[12] In our study, 73 (16.0%) calls were received for trauma and out of them only 23 (16.8%) required operative intervention while 4 (3%) were admitted for observation in general surgical unit. Other patients were either discharged or referred to other units concerned. A study by Dattani *et al*.[13] showed that general surgeons assessed 30.1% trauma call patients; only 12.3% patients were admitted under the general surgeons. 9.6% patients required operative surgical intervention, while 2.7% patients were admitted for observations. In another study,[12] trauma comprised approximately 2% of the overall general surgical emergency workload in which general surgeons were involved in the assessment of 25% of severely injured patients, out of which less than 10% patients needed surgery. We recommend that A and E staff should be competent, well trained, and made responsible for initial management of trauma patients. Following primary and secondary surveys, the appropriate teams should be called but in penetrating abdominal and thoracic injuries, presence of a general surgeon remained crucial.

A study[14] performed to find out the ‘Unseen’ on-call workload of a general surgical team showed that up to 5.5 hours per day on-call was spent assessing referrals. The A and E Department referred 46% of patients of which only 7% required surgical management. This study showed that while hours of work are important in assessing the workload of a resident on-call, the intensity of the workload is just as important in determining the impact on staff. Another audit of general practitioner referrals to an acute surgical unit showed that during 6 months duration, a total of 78 admissions were considered inappropriate of whom 23 patients were thought to have needed neither surgical admission nor opinion.[15] These studies have reinforced our observation that there is a greater workload than revealed by audit of just surgical admissions and operations alone.

Apart from examining the on-call workload of surgical residents, an assessment of on-call activity is needed to maximize educational merit of our surgical residents. Morton *et al*.[16] reported on-call night activity of surgical resident and concluded that it consists primarily of activities of daily living, patient evaluation, and communication. Sleep accounts for nearly one third of all on-call activity.

Tendulkar *et al*.[17] reported that when heart rate is used as an indicator of combined physiologic and psychologic stress, surgical residents achieve stress levels of tachycardia “on call.” Surgical residents also exhibit an increase in circulating WBC count “on call.” By burdening the surgical resident in doing work not required for their academic or research work, this stress level was increased much more and inappropriately, most probably experienced by our surgical residents.

## CONCLUSIONS

The major workload of an on-call surgical emergency team deals with the acute conditions of abdomen with appendicectomy being the most frequent operation performed. A substantial proportion of patients, however, suffer from other conditions including trauma that require a holistic approach to care and a wide range of skills and experience that may cross subspecialty and specialty divisions.

The results of this study are helpful in planning better emergency service delivery to patients and in focusing and improving the training of surgical residents. There is a need for a structured training program for emergency surgery of general surgical residents specially revolving around these common pathologies and their operative managements. Government at various levels should provide modern diagnostic tools for the accurate preoperative diagnosis of surgical emergencies in tertiary care public hospitals. These measures will help to improve the management and outcome of surgical emergencies.
